# Supplementary material for: Critical role of biomass burning aerosols in enhanced historical Indian Ocean warming
Source: Nat Commun. 2023 Jun 14;14:3508. doi: 10.1038/s41467-023-39204-y (PMC10267193; doi:10.1038/s41467-023-39204-y)
Supplement: Supplementary file 1 — Supplementary Information [file 41467_2023_39204_MOESM1_ESM.pdf]

Supplementary Information for:

Critical role of biomass burning aerosols in enhanced historical Indian Ocean warming

Yiqun Tian[1], Shineng Hu[1*], Clara Deser[2]

[1] *Division of Earth and Climate Sciences, Nicholas School of the Environment, Duke University, Durham, NC, USA*

[2] *Climate and Global Dynamics, National Center for Atmospheric Research, Boulder, CO, USA*

[*]**Corresponding author:** Shineng Hu (shineng.hu@duke.edu)

Division of Earth and Climate Sciences, Nicholas School of the Environment, Duke University

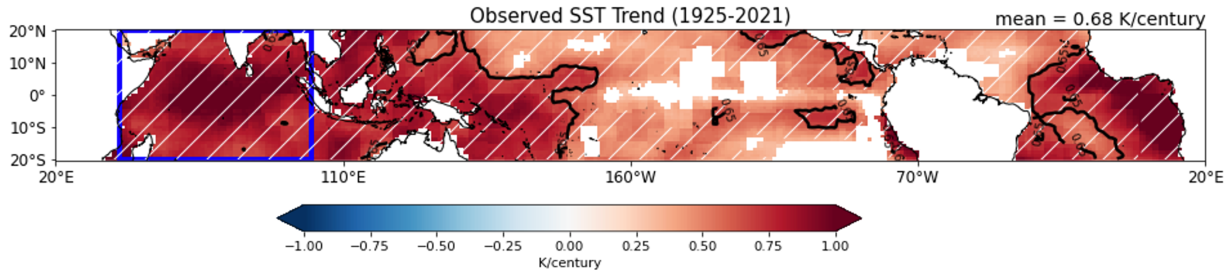

Supplementary Figure 1 | Observed tropical sea surface temperature (SST) trends since 1925. Observed tropical SST trends (K/century) during 1925-2021, averaged across four SST datasets (ERSSTv5, HadISST, COBE-SST, Kaplan SST). The black contour line represents the isopleth of the tropical-mean SST trend (value shown on the top-right corner). This figure is the same as Fig. 1a but with only the regions where all four datasets agree on the sign of the SST trend being shown. White hatches represent the regions that are 99% significant based on a t-test.

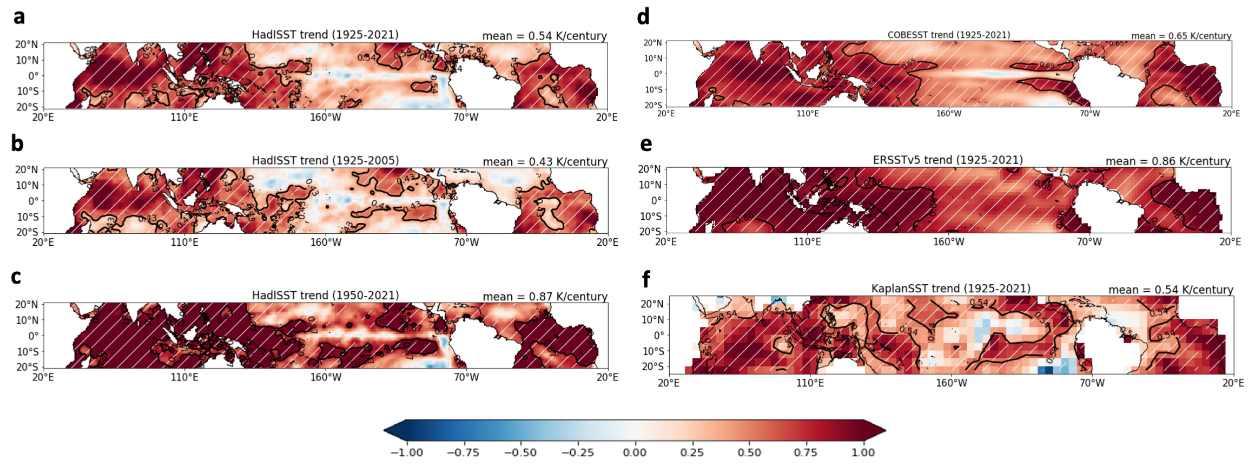

Supplementary Figure 2 | Observed tropical sea surface temperature (SST) trends in different datasets. a-c, SST trends in HadISST during the periods of **a** 1925-2021, **b** 1925-2005, and **c** 1950-2021. **d-f,** SST trends during 1925-2021 for **d** ERSST v5, **e** COBE-SST, and **f** Kaplan SST v2. The black contour line represents the isopleth of the tropical-mean SST trend (value shown on the top-right corner). White hatches in **a**-**f** represent the regions that are 99% significant based on a t-test.

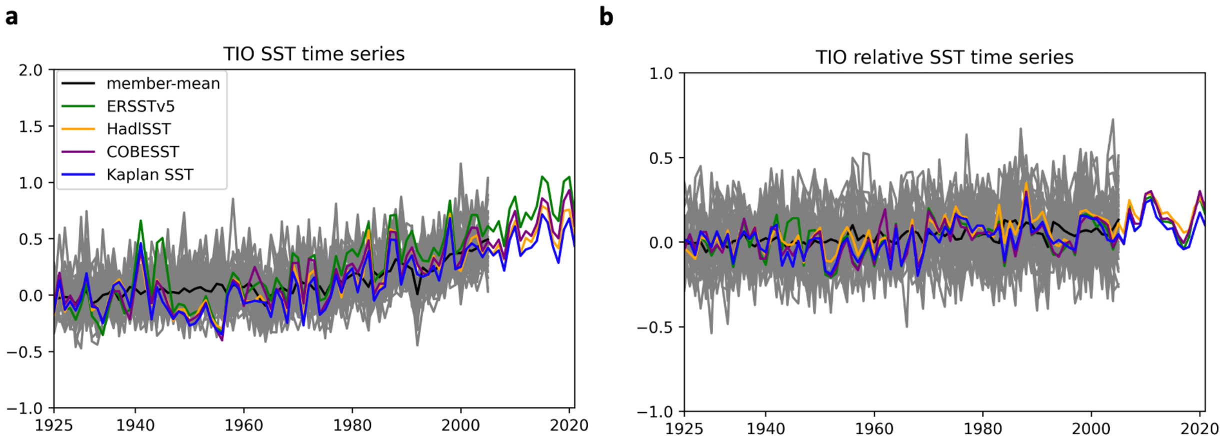

Supplementary Figure 3 | Observed and model simulated variations in annual-mean tropical Indian Ocean (TIO) sea surface temperature (SST) since 1925. a, TIO absolute SST (K) and **b,** TIO relative SST (K) with respect to the reference period 1925-1944. In both panels, the grey lines are for each Community Earth System Model v1 (CESM1)-Large Ensemble (LE) all-forcing ensemble member and the black line is for ensemble-mean in the all-forcing experiments, and the colored lines are for different observational SST datasets. Relative SST is defined as absolute SST minus tropical-mean SST.

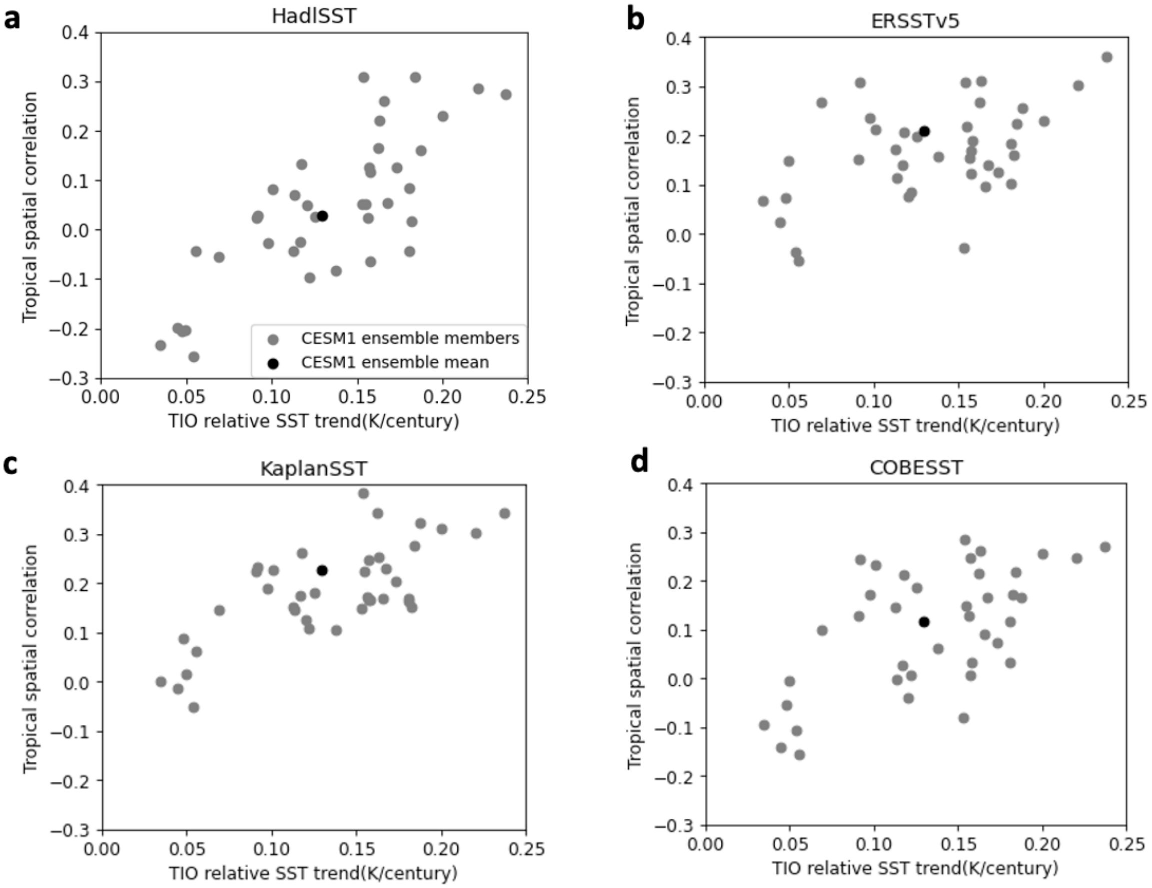

Supplementary Figure 4 | Model simulated tropical sea surface temperature (SST) trend pattern against observations. In all panels, the horizontal axis is for tropical Indian Ocean (TIO) relative SST trends during 1925-2005, and the vertical axis is for the pattern correlation of tropical SST trends within 20°S-20°N between an individual ensemble member (grey) or ensemble-mean (black) of Community Earth System Model v1 (CESM1)-Large Ensemble (LE) all-forcing experiments and observations. The only difference among the four panels is the observational dataset. **a,** HadISST. **b,** ERSSTv5. **c**, Kaplan SST v2. **d**, COBESST V2. Relative SST is defined as absolute SST minus tropical-mean SST.

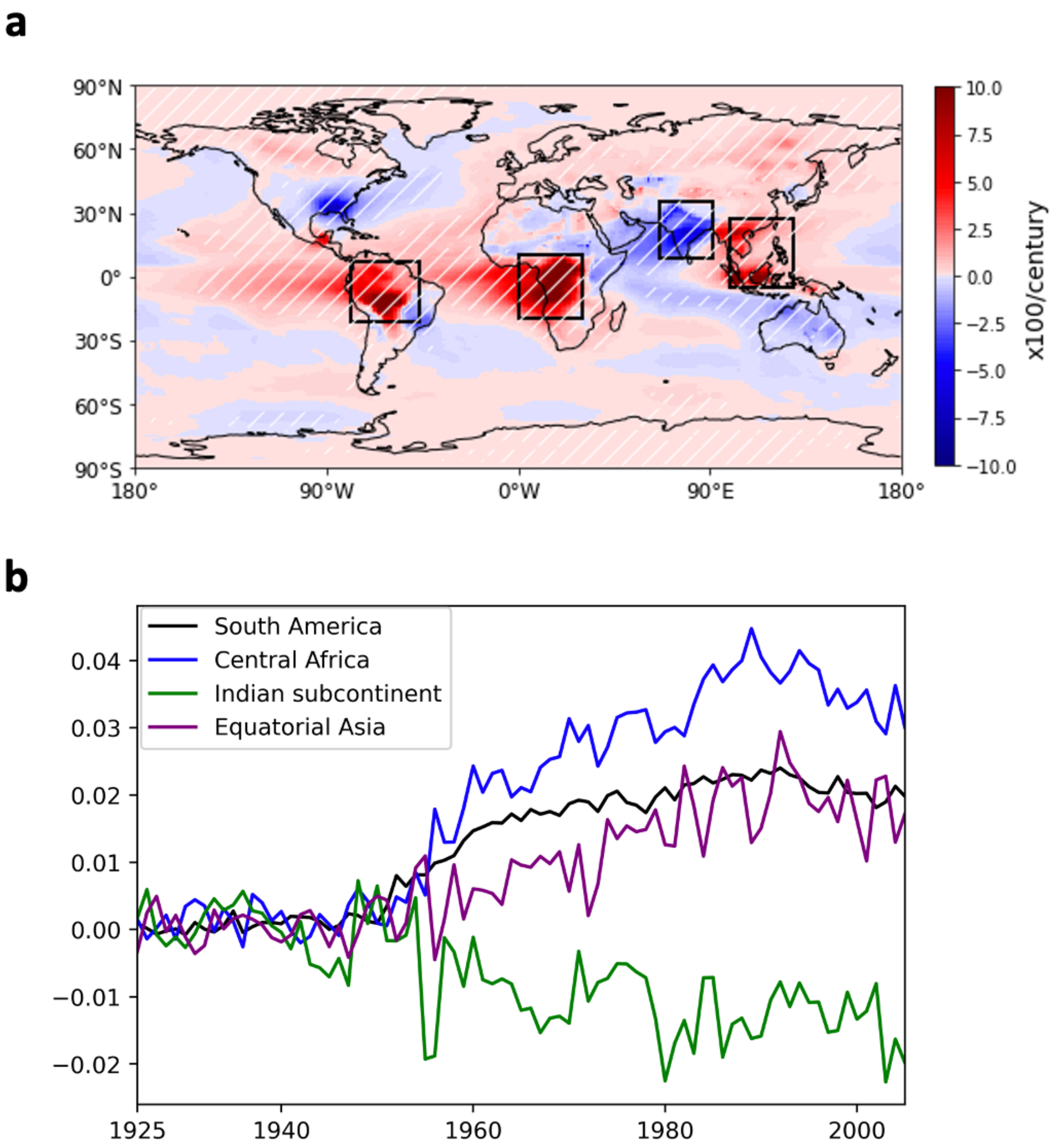

Supplementary Figure 5 | Biomass burning (BMB) aerosol changes in the historical period. **a,** Aerosol optical depth (AOD) trends during 1925-2005 associated with BMB aerosol changes. **b,** AOD variations in specific regions associated with BMB aerosol changes. Boxes used for averaging AOD are shown in **a**. White hatches in **a** represent the regions that are 99% significant based on a t-test.

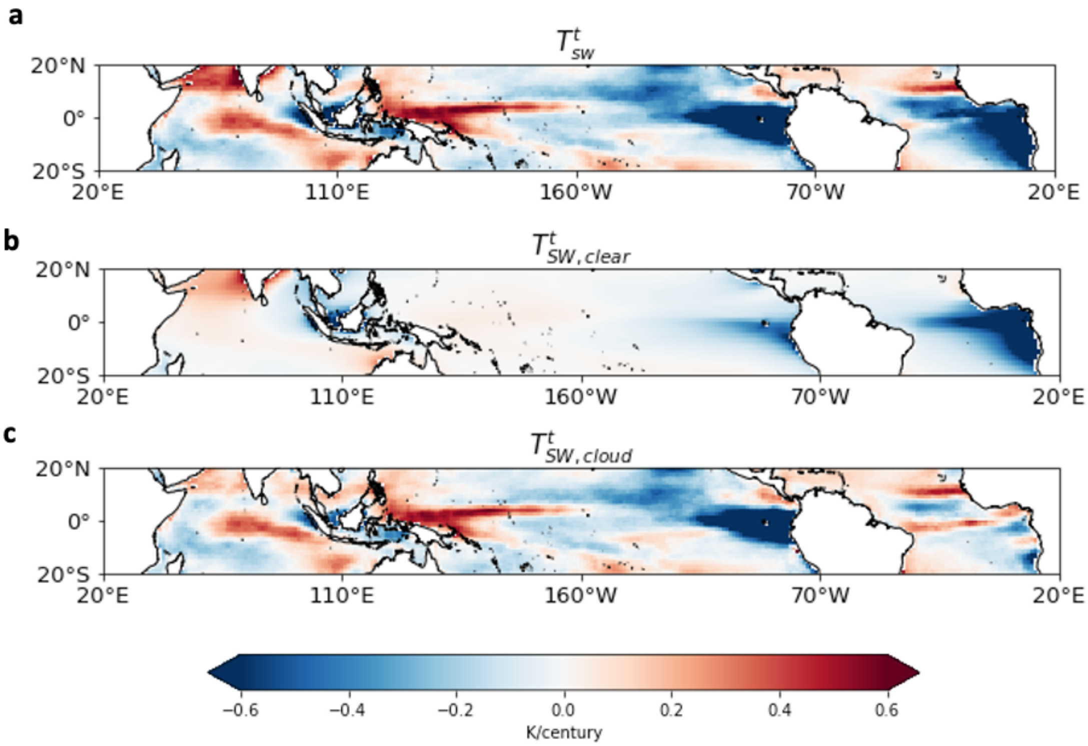

Supplementary Figure 6 | Biomass burning (BMB) aerosols-induced tropical sea surface temperature (SST) trends. BMB-induced tropical SST trends (K/century) during 1925-2005 associated with **a** total (T^t_{SW}), **b** clear-sky ($T^t_{SW,clear}$), and **c** cloud ($T^t_{SW,cloud}$) shortwave radiative flux changes.

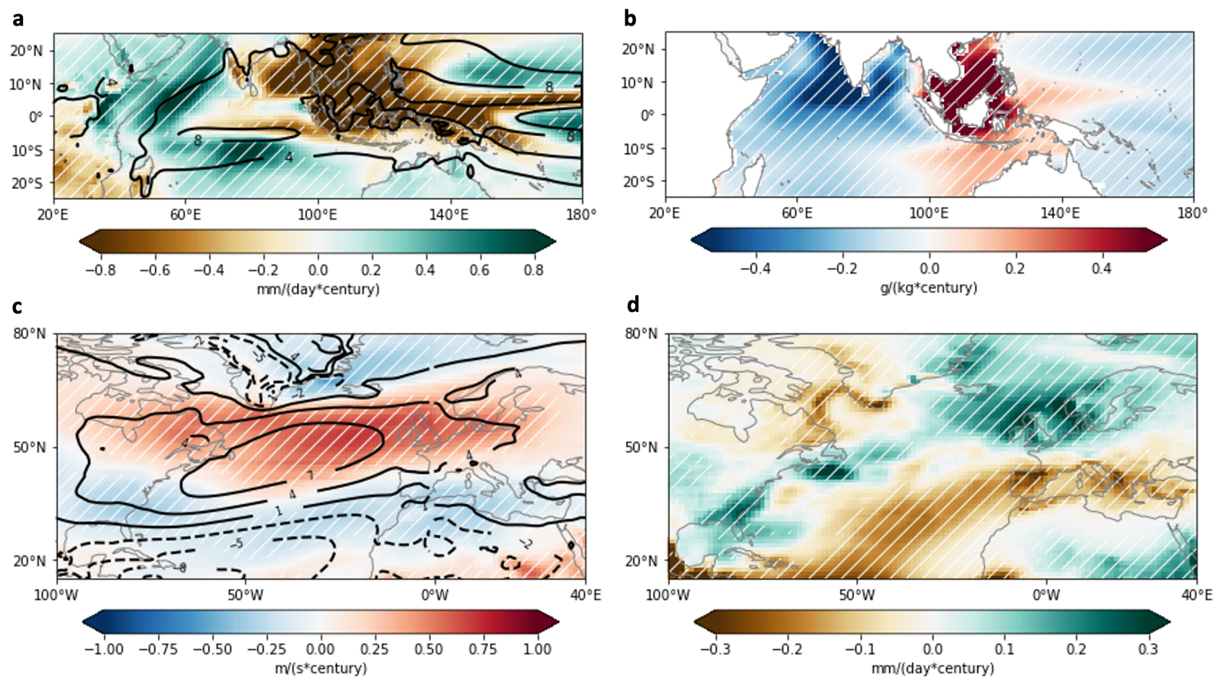

Supplementary Figure 7 | Climate changes simulated by Community Earth System Model v1 (CESM1)-Large Ensemble (LE) all-forcing experiments. **a**, Ensemble-mean rainfall trends (colors; mm/d/century) overlaid by climatological rainfall (contours; mm/d) and **b**, Ensemble-mean ocean salinity trends (g/kg/century) during 1925-2005. **c**, Ensemble-mean 850 mb zonal wind trends (colors; m/s/century) overlaid by climatological 850 mb zonal wind (contours; m/s) and **d**, Ensemble-mean rainfall trends (mm/d/century) during 1925-2005. White hatches in **a-d** represent the regions that are 99% significant based on a t-test.

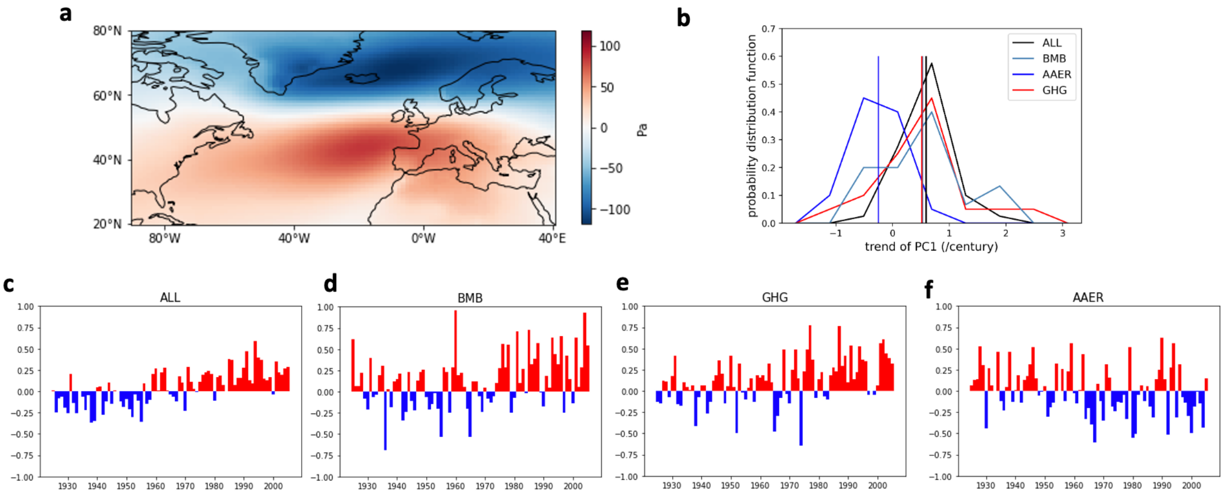

Supplementary Figure 8 | North Atlantic Oscillation (NAO) changes in the single-forcing Community Earth System Model v1 (CESM1)-Large Ensemble (LE). a, Sea level pressure anomalies associated with the positive phase of NAO. **b**, Probability distribution functions of NAO trends across all the members in each set with vertical lines highlighting the ensemble averages. **c-f**, Ensemble-mean NAO series in all-forcing (ALL), biomass burning aerosols (BMB), greenhouse gas (GHG), anthropogenic aerosols (AAER), respectively.

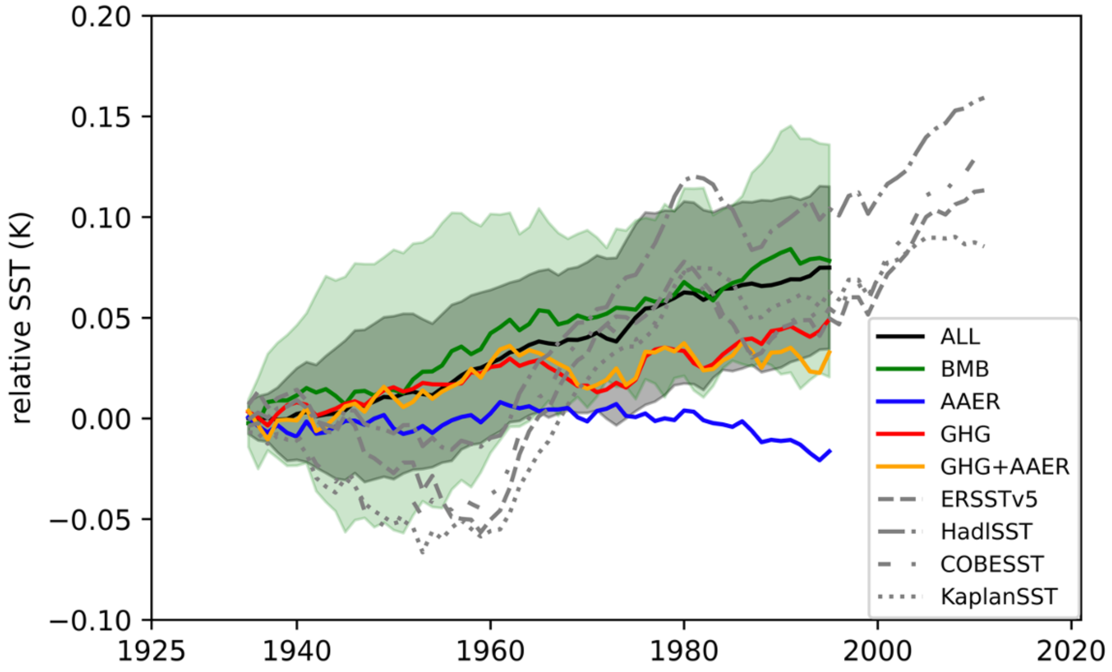

Supplementary Figure 9 | Observed and model simulated tropical Indian Ocean (TIO) relative sea surface temperature (SST) series. a, 21-year running mean for TIO relative SST variations (°C) since 1920 for observations (grey lines) and ensemble means from the single-forcing Community Earth System Model v1 (CESM1)-Large Ensemble (LE); see Methods for details. The grey and green shadings are one standard deviation among ensemble members for all-forcing (ALL) and biomass burning (BMB), respectively.
